# A portable epigenetic switch for bistable gene expression in bacteria

**DOI:** 10.1038/s41598-019-47650-2

**Published:** 2019-08-02

**Authors:** David R. Olivenza, Hervé Nicoloff, María Antonia Sánchez-Romero, Ignacio Cota, Dan I. Andersson, Josep Casadesús

**Affiliations:** 1 Departamento de Genética, Facultad de Biología, Universidad de Sevilla, Apartado 1095, 41080 Sevilla, Spain; 2 Department of Medical Biochemistry and Microbiology, Uppsala University, 751 23 Uppsala, Sweden; 3 Present Address: Centre for Research in Agricultural Genomics, CSIC-IRTA-UAB-UB, Campus UAB, Bellaterra, 08193 Barcelona, Spain

**Keywords:** Bacterial synthetic biology, DNA methylation

## Abstract

We describe a portable epigenetic switch based on *opvAB*, a *Salmonella enterica* operon that undergoes bistable expression under DNA methylation control. A DNA fragment containing the *opvAB* promoter and the *opvAB* upstream regulatory region confers bistability to heterologous genes, yielding OFF and ON subpopulations. Bistable expression under *opvAB* control is reproducible in *Escherichia coli*, showing that the *opvAB* switch can be functional in a heterologous host. Subpopulations of different sizes can be produced at will using engineered o*pvAB* variants. Controlled formation of antibiotic-resistant and antibiotic-susceptible subpopulations may allow use of the *opvAB* switch in the study of bacterial heteroresistance to antibiotics.

## Introduction

Biosensors able to detect environmental signals are made of a sensor that detects a given input and a reader that responds to the input generating a detectable signal in a quantitative or semi-quantitative fashion^1^. Classical sensors employ enzymes or whole cells. Enzyme-based biosensors present the advantage of high selectivity but the need for purification can be a drawback due to technical difficulties and high cost. In contrast, whole-cell sensors are often easy to use and inexpensive, especially if microbial strains are used^2^. A common type of microbial biosensor is an engineered strain that responds to physical or chemical inputs generating electrochemical or optical signals. Sensors of this type often employ a promoter sensitive to a specific input and a reporter gene that produces a detectable signal^1,3^. The literature contains multiple examples of sensors that detect electrochemical and optical signals, and use of fluorescent proteins has become widespread in the last decade^4^.

An alternative to genetic circuits able to process information in living cells is the design of epigenetic switches. This approach has received special attention to develop diagnostic tests for human diseases^5–7^, while synthetic biology based on bacterial epigenetics remains largely unexplored. A relevant exception is the recent development of biosensors based on DNA adenine methylation using *Escherichia coli* as host^8^.

**Figure 1:**
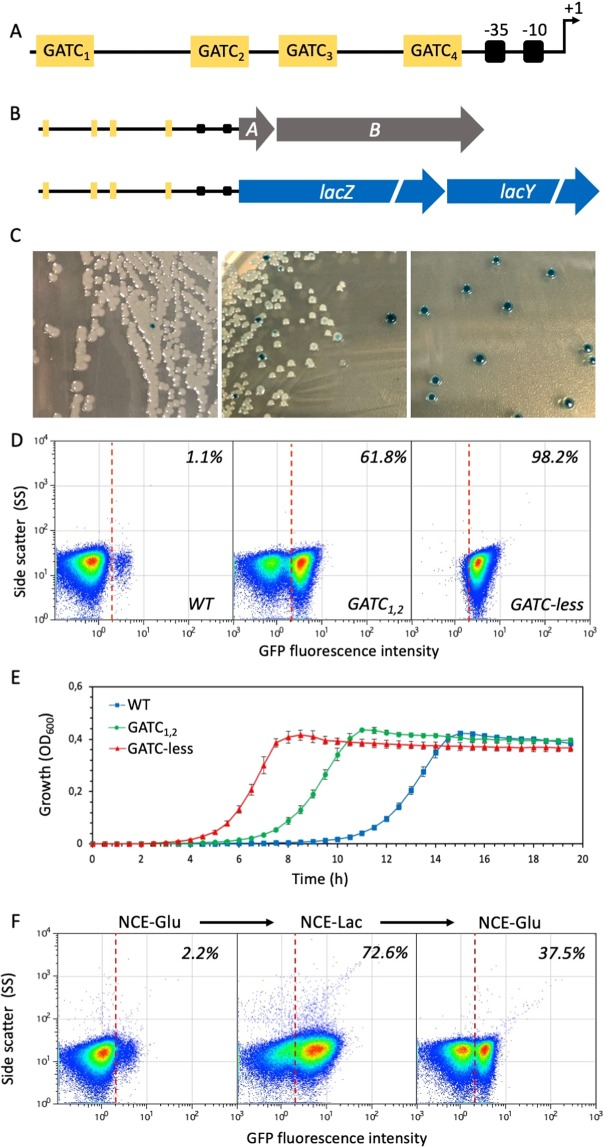
Formation of Lac^OFF^ and Lac^ON^ subpopulations under *opvAB* control. (**A**) Diagram of the *opvAB* promoter and regulatory region, with the GATC sites outlined. (**B**) Diagram of the wild type *opvAB* operon and the P_*opvAB*_::*lacZY* construct. (**C**) Colonies formed on LB + X-gal by a *S*. *enterica* strain carrying the *lacZY* operon under the control of the wild type *opvAB* control region (SV9700, left), and by *S*. *enterica* strains carrying the *lacZY* operon under the control of mutant *opvAB* control regions (SV9701, P_*opvAB*_ GATC_1,2_::*lacZY*::*gfp*, center; SV9702, P_*opvAB*_ GATC-less::*lacZY*::*gfp*, right). (**D**) Flow cytometry analysis of P_*opvAB*_::*lacZY* expression in strains SV9700, SV9701 and SV9702. The sizes of Lac^ON^ subpopulations are indicated. (**E**) Growth of strains SV9700, SV9701 and SV9702 in NCE-lactose. Error bars represent the standard error of the mean from 3 independent replicates. (**F**) Reversible formation of Lac^OFF^ and Lac^ON^ subpopulations under *opvAB* control in strain SV9700.

In this study, we describe the construction and application of an epigenetic switch that drives gene expression in a bistable fashion. Bistability generates bacterial subpopulations that differ in a specific phenotypic trait (e. g., antibiotic resistance) and have defined sizes. The switch is based on *opvAB*, a bacterial operon subjected to epigenetic control by DNA adenine methylation^9–11^. Transcription of *opvAB* is bistable, with concomitant formation of OpvAB^OFF^ and OpvAB^ON^ cells^9^. Bistability is controlled by binding of the OxyR transcription factor to a regulatory region upstream of the *opvAB* promoter (Fig. 1A)^10^. This region contains four sites for OxyR binding and four GATC motifs. OpvAB^OFF^ and OpvAB^ON^ cell lineages display alternative patterns of OxyR binding, which in turn cause alternative patterns of GATC methylation: in the OFF state, GATC_2_ and GATC_4_ are methylated; in the ON state, GATC_1_ and GACT_3_ are methylated^10^. Here, we show that a cassette of 689 nucleotides containing the *opvAB* promoter and the upstream regulatory region confers bistability to heterologous genes, and describe examples of *opvAB*-based constructs that produce bacterial subpopulations with distinct phenotypes. One of the examples involves formation of an antibiotic-resistant subpopulation upon cloning of an antibiotic resistance gene downstream of the *opvAB* promoter. This construct may provide an experimental system to study bacterial heteroresistance (HR) to antibiotics under highly controlled conditions^12^. HR is a phenotype where a bacterial isolate is characterized by the presence of a main susceptible population and a subpopulation with higher antibiotic resistance. Increasing evidence suggests that heteroresistance can lead to treatment failure^12–17^. Yet, little is known regarding the characteristics of the heteroresistance phenotypes (i.e. the size of the resistant subpopulation or its level of resistance) that are linked to treatment failure. Animal experiments, where infections are started with bacterial cultures that carry an antibiotic resistance gene under control of the *opvAB* switch, would allow control of the frequency of the resistant subpopulation and determination of how different ratios of resistant:susceptible bacteria influence treatment outcome^17^. Other potential uses of the *opvAB* switch in synthetic biology are discussed.

## Results

### Bistable expression of *lacZY* under *opvAB* transcriptional control

The ability of the *opvAB* epigenetic switch to confer bistable expression to a heterologous locus was tested by engineering a strain that harbored the *E*. *coli lacZY* operon downstream of the *opvAB* promoter and its upstream regulatory region (P_*opvAB*_ - Fig. 1A,B). To avoid cell-to-cell heterogeneity associated with variations in plasmid copy number, the construct was engineered in the *S*. *enterica* chromosome. Construction involved replacement of the *opvAB* coding region with a promoterless *lacZY* operon, leaving the *opvAB* promoter and upstream regulatory region intact. The construct harbored the *opvA* ribosome binding site (RBS). Plating of the engineered strain on LB containing X-gal yielded Lac^+^ (blue) and Lac^−^ (white) colonies, thus revealing bistable expression (Lac^OFF^ or Lac^ON^) of the heterologous *lacZY* operon (Fig. 1C). Streaking of either Lac^+^ or Lac^−^ colonies on X-gal agar yielded a mixture of Lac^+^ and Lac^−^ colonies, thus indicating the occurrence of reversible bistability (“phase variation”) as previously described for the native *opvAB* locus^9^.

Calculation of phase variation frequencies indicated a frequency of 1.1 × 10^−4^ ± 0.3 per cell and generation for the OFF→ON transition, and 3.4 ± 0.1 × 10^−2^ per cell and generation for the ON→OFF transition. The 300-fold difference between switching rates was two-fold lower than in the native *opvAB* locus (OFF → ON, 6.1 ± 1.7 × 10^−5^; ON → OFF 3.7 ± 0.1 × 10^−2^; 600-fold difference between switching rates)^9^. The increased size of the Lac^ON^ subpopulation may result from multiple factors including potential differences in mRNA stability and codon usage constraints.

Variants of the P_*opvAB*_::*lacZY* construct were engineered to further explore the ability of *opvAB-*driven transcription to confer bistable expression to a heterologous locus. One such variant involved the use of a mutant *opvAB* regulatory region lacking GATC sites 1 and 2 (GATC_1,2_), previously shown to increase the size of the OpvAB^ON^ subpopulation^10^. As expected, a higher proportion of Lac^+^ colonies was detected (Fig. 1C). Another variant, used as control, lacked all *opvAB* GATC sites (GATC-less) and locked *lacZY* transcription in the ON state (Fig. 1C) as previously described for the native *opvAB* operon^9^.

Variants carrying a green fluorescent protein gene (*gfp*) dowstream of the *lacZY* operon were also engineered, and assessment of subpopulation sizes by flow cytometry confirmed that the Lac^ON^ subpopulation formed by the wild type *opvAB* switch was smaller than that formed by the GATC_1,2_ variant (Fig. 1D). Furthermore, only cells in the Lac^ON^ state were detected in the strain that harbored the GATC-less construct, and subpopulation formation was abolished as above (Fig. 1D).

The ability of the *opvAB* switch to permit selection of one of the subpopulations was examined by testing the ability of strains carrying P_*opvAB*_::*lacZY*::*gfp* and P_*opvAB*_ GATC_1,2_::*lacZY*::*gfp* constructs to grow in minimal medium with lactose as sole carbon source. As above, a strain carrying the GATC-less P_*opvAB*_::*lacZY*::*gfp* construct was included as a control. Assessment of the growth patterns of these strains revealed that the time required for culture saturation was dependent on the size of the Lac^ON^ subpopulation present at the start of the culture (Fig. 1E). Reversibility of the Lac^ON^ state was confirmed by growth on NCE-glucose (Fig. 1F).

### Bistable expression of the chimaeric *opvAB*::*lacZY* operon in a heterologous host, *E*. *coli*

**Figure 2:**
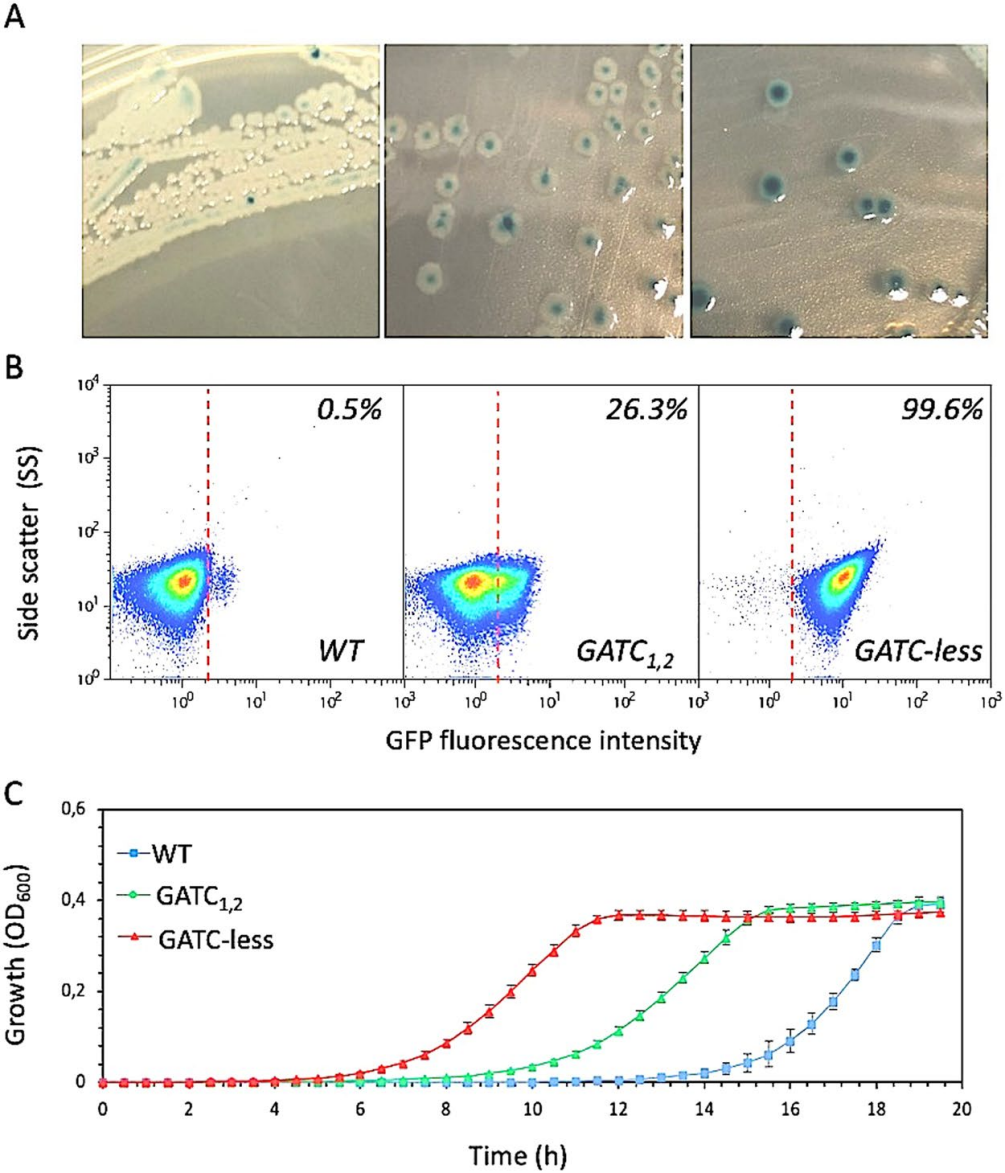
Formation of Lac^OFF^ and Lac^ON^ subpopulations under *opvAB* control in *E*. *coli*. (**A**) Left: Colonies formed on LB + X-gal by an *E*. *coli* strain carrying the *lacZY* operon under the control of the wild type *opvAB* control region (strain DR22). Center and right: Colonies formed on LB + X-gal by *E*. *coli* strains carrying the *lacZY* operon under the control of mutant *opvAB* control regions (DR23 and DR24). (**B**) Flow cytometry analysis of P_*opvAB*_::*lacZY* expression in strains DR22, DR23 and DR24. The sizes of Lac^ON^ subpopulations are indicated. (**C**) Growth of strains DR22, DR23 and DR24 in NCE-lactose. Error bars represent the standard error of the mean from 3 independent replicates.

The functionality of the *opvAB* switch in a heterologous host was tested in *E*. *coli*. For this purpose, the P_*opvAB*_::*lacZY*::*gfp* construct and its GATC_1,2_ and GATC-less variants were introduced into the chromosome of *E*. *coli* DR3 (Δ*lacZY*). Strains carrying the P_*opvAB*_::*lacZY-gfp* and P_*opvAB*_ GATC_1,2_::*lacZY*::*gfp* constructs (DR22 and DR23, respectively) formed Lac^+^ and Lac^–^ colonies on X-gal agar, and the number of Lac^+^ colonies was higher in the strain carrying the P_*opvAB*_ GATC_1,2_::*lacZY*::*gfp* construct. The strain carrying the GATC-less construct (DR24) formed Lac^+^ colonies only (Fig. 2A). Flow cytometry assessment of GFP expression upon growth in LB confirmed the occurrence of subpopulations of Lac^OFF^ and Lac^ON^ cells in the strains carrying the P_*opvAB*_::*lacZY*::*gfp* and P_*opvAB*_ GATC_1,2_::*lacZY*::*gfp* constructs but not in the strain carrying the GATC-less construct (Fig. 2B). As above, growth pattern assessment revealed that the time required for culture saturation was dependent on the initial size of the Lac^ON^ subpopulation (Fig. 2C). Altogether, these observations indicated that the *opvAB* switch is functional in *E*. *coli*.

### Bistable expression of antibiotic resistance genes under *opvAB* control

**Figure 3:**
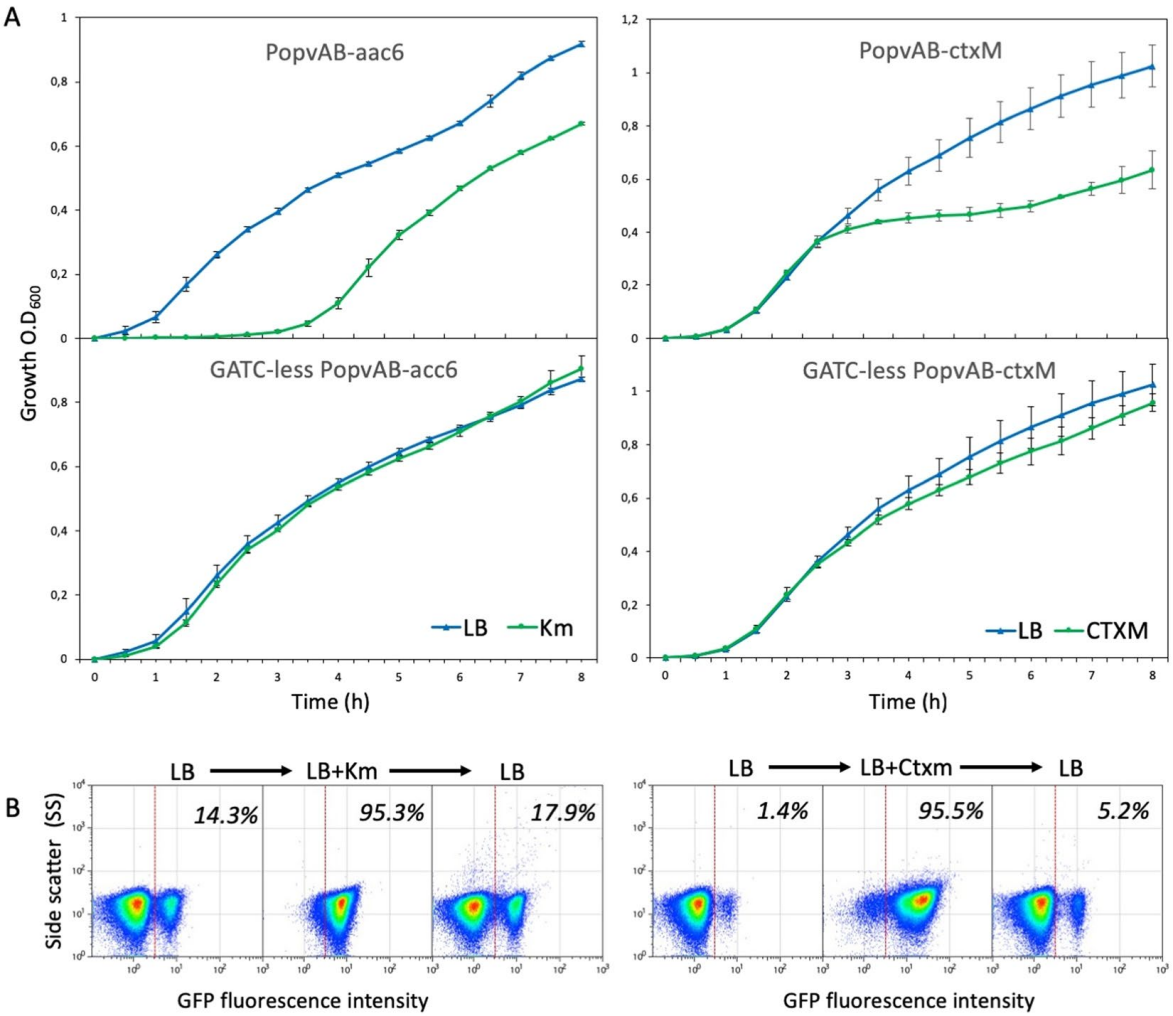
(**A**) Growth of strains SV9703 (P_*opvAB*_*::aac6*::*gfp*), SV9705 (P_*opvAB*_ GATC-less::*aac6*::*gfp*) SV9706 (P_*opvAB*_::*ctxM*::*gfp***)** and SV9707 (P_*opvAB*_ GATC-less::*ctxM*::*gfp*) in LB and in LB + antibiotic (kanamycin and cefotaxime, respectively). (**B**) Left: reversibility of formation of Km^s^ and Km^r^ subpopulations under *opvAB* control. Right: reversibility of formation of CtxM^s^ and CtxM^r^ subpopulations under *opvAB* control.

An additional test of the ability of the *opvAB* bistable switch to generate bacterial subpopulations was performed by cloning antibiotic resistance genes downstream of the *opvAB* promoter in the *S*. *enterica* chromosome. The antibiotic resistance genes chosen for these experiments were *aac3-Ib* (henceforth, *aac3)* and *aac(6*′*)-Ib-cr* (henceforth, *aac6*), which encode aminoglycoside acetyl transferases^18^, and *bla*_CTX-M-15_ (henceforth, *ctxM)*, which encodes an extended-spectrum β-lactamase^19^. In these constructs, the native ribosome binding sites were replaced with a stronger RBS, named BI^20^ to adjust the sensitivity of the switch to a level that could permit unambiguous detection of the antibiotic resistance phenotype under study, thus facilitating discrimination between OFF and ON cells. Experiments with strains carrying P_*opvAB*_::*aac6*::*gfp* and P_*opvAB*_::*ctxM*::*gfp* fusions (strains SV9703 and SV9706, respectively) yielded bacterial subpopulations resistant to kanamycin and to cefotaxime, respectively (Fig. 3A). Controls using strains that constitutively expressed *aac6* and *ctxM* (SV9705 and SV9707, respectively) showed that the concentrations of antibiotics used permitted growth (Fig. 3A). The wild type strain ATCC 14028 failed to grow under such conditions, confirming that the concentrations of antibiotics used were bactericidal.

Flow cytometry analysis confirmed that growth in the presence of kanamycin and cefotaxime was a consequence of subpopulation selection (Fig. 3B), excluding the idea that growth might result from selection of mutants present in the inoculum. This conclusion was further strengthened by the observation that growth in LB restored the initial sizes of ON and OFF subpopulations (Fig. 3B).

### Use of the OpvAB synthetic switch in generating antibiotic heteroresistance

**Figure 4:**
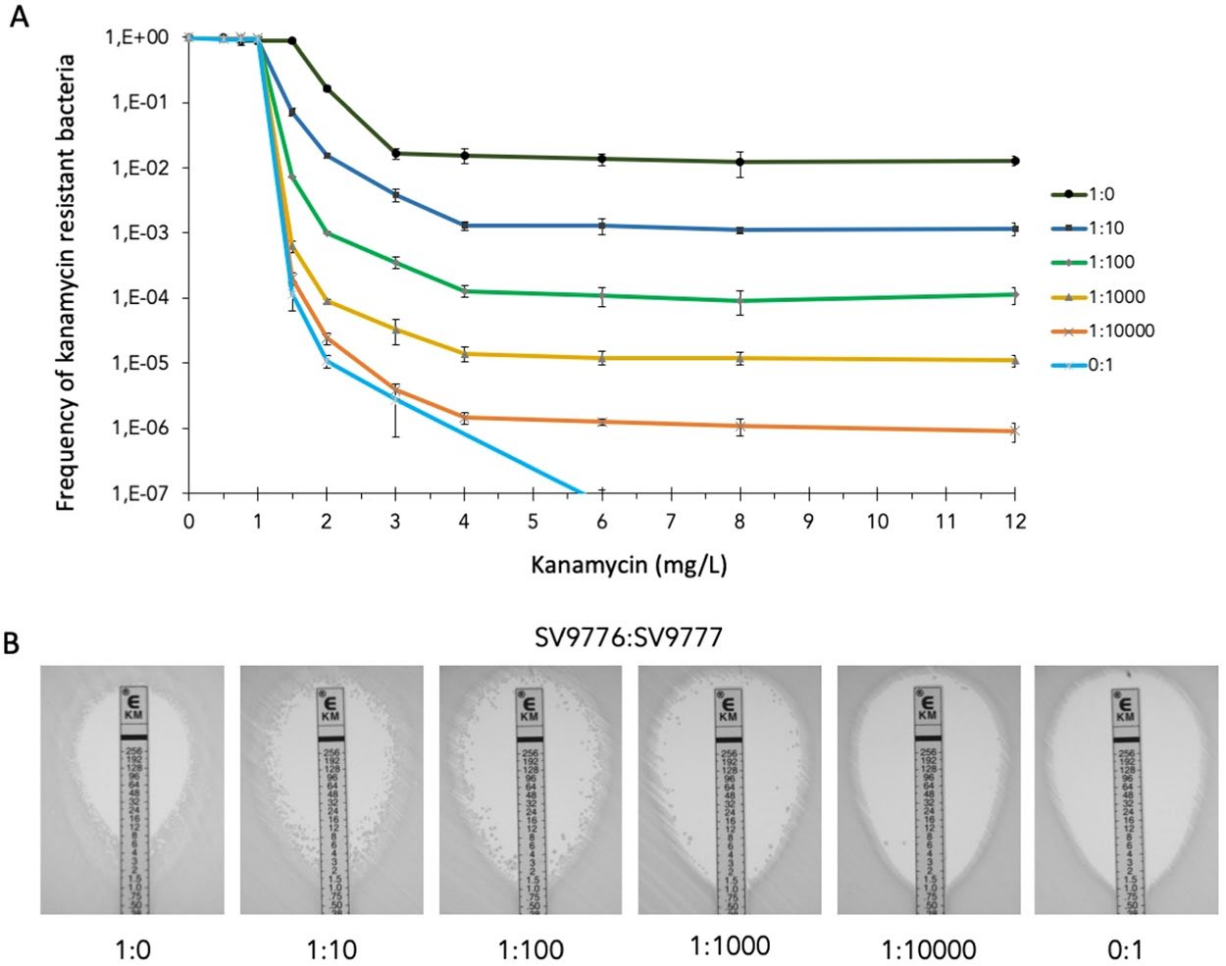
The *opvAB* switch as a tool for the study of antibiotic heteroresistance. (**A**) Population analysis profile (PAP) tests of kanamycin resistance in strains SV9776 and SV9777 (carrying P_*opvAB*_::BI-*aac3*::*gfp* and P_*opvAB*_::*gfp* constructs, respectively). The proportions indicated are those of the mixtures of SV9776:SV9777. Error bars represent standard deviations of three independent mixtures. (**B**) Epsilometer tests (Etests) performed on the same mixtures of SV9776 and SV9777. The kanamycin resistant subpopulations appear as low-density lawns (mixtures 1:0 and 1:10) or as isolated colonies growing at kanamycin concentrations above the MIC of the main susceptible population ([Km] > 1 mg/L - mixtures 1:10 to 1:10,000). No kanamycin resistant subpopulation exists for the 0:1 mixture.

As a proof of concept, we examined the utility of the OpvAB switch to address antibiotic heteroresistance and the question of what proportions of resistant subpopulations might lead to clinical treatment failure. Specifically, we tested whether the OpvAB switch could generate, in a susceptible main population, defined subpopulations of cells with increased antibiotic resistance. For this purpose, we used a *S*. *enterica* strain harboring a P_*opvAB*_::BI-*aac3*::*gfp* construct (SV9776). Expression of *aac3* (Aac3^ON^) leads to kanamycin resistance (Km^r^). The frequency of Km^r^ cells formed by a pure culture of SV9776 was 1 × 10^−2^ (Fig. 4A), similar to the frequency of ON cells detected when *gfp* was cloned behind the *opvAB* promoter (1.1%: Fig. 1D). To obtain smaller subpopulation sizes without altering other phenotypic traits of the strain, SV9776 was mixed with an isogenic strain that expressed P_*opvAB*_::*gfp* (SV9777) and did not produce any Km^r^ resistant subpopulation. Mixtures of cells were prepared from overnight cultures in Mueller-Hinton (MH) broth at proportions 1:10, 1:100, 1:1,000, 1:10,000 and 0:1. Population analysis profile (PAP) tests were then performed by plating on MH agar containing increasing concentrations of kanamycin. After overnight incubation, the number of resistant cells and total number of cells were determined to allow calculation of the fraction of resistant cells. The numbers of Km^r^ colonies detected in the PAP tests were proportional to the amounts of the Aac3^ON^ subpopulations present in each mixture, and ranged from 1 × 10^−2^ to 1 × 10^−6^ (Fig. 4A). Epsilometer tests (Etests) further confirmed that the size of the Km^r^ subpopulation decreased in a manner proportional to dilution (Fig. 4B).

## Discussion

In its native host, the *opvAB* operon undergoes bistable transcription, which generates OpvAB^ON^ and OpvAB^OFF^ subpopulations^9^. Bistability is reversible (“phase-variable”) and the switching rate is skewed to OFF in the wild type^9,11^. In this study, we show that a 689 bp DNA fragment containing the *opvAB* promoter and the *opvAB* upstream activating sequence (UAS) confers bistability to genes cloned downstream. For instance, an engineered P_*opvAB*_::*lacZY* operon produces Lac^OFF^ and Lac^ON^ subpopulations (Fig. 1C), and addition of a *gfp* reporter gene permits discrimination of Lac^OFF^ and Lac^ON^ cells by flow cytometry (Fig. 1D). Utilization of L-lactose sustains growth of Lac^ON^ cells (Fig. 1E), thereby producing increased fluorescence. However, because the *opvAB* switch is reversible, in the absence of L-lactose the system slowly returns to its initial state, with a strong predominance of Lac^OFF^ cells (Fig. 1F).

The fact that the *opvAB* cassette is functional in *E*. *coli* (Fig. 2) suggests that the switch can be used to generate bistability in other heterologous hosts. However, the need of both Dam methylation and OxyR may be an obvious limitation. Aside from this caveat, the versatility of the switch is reinforced by an additional example of subpopulation formation presented in Fig. 3: P_*opvAB*_-driven bistable expression of kanamycin and cefotaxime resistance genes permitted selection of antibiotic-resistant subpopulations in a reversible fashion.

Introduction of mutations in the upstream regulatory region of the native *opvAB* operon alters the switching rate, yielding OpvAB^ON^ and OpvAB^OFF^ subpopulation sizes that are different from those of the wild type^10,11^. Hence, variants of the *opvAB* switch can be engineered to modulate subpopulation sizes at will. For instance, a variant (GATC_1,2_) that lacks two of the four GATC sites present in the wild type increases the initial size of the ON subpopulation (Figs 1 and 2). Additional UAS variants that yield subpopulations of different sizes have been described^10^, and their use may allow choice of other switching frequencies. Modification of the ribosome-binding site of genes under *P*_*opvAB*_ control can also contribute to adjust the sensitivity of the switch, facilitating detection of the phenotype under study. For instance, use of the BI ribosome binding site^20^ permitted unambiguous detection of *aac3*-mediated kanamycin resistance, thereby facilitating discrimination of Km^r^ cells (Fig. 4).

As a proof of concept, we have used the *opvAB* switch to produce antibiotic-resistant and antibiotic-susceptible bacterial subpopulations of predetermined sizes. The aim of these experiments was to mimic under laboratory conditions bacterial heteroresistance to antibiotics, a phenomenon where small subpopulations of cells show higher antibiotic resistance than the main population^12^. Heteroresistance is difficult to detect and study in clinical samples^12^, and accurate assessment of the frequencies of subpopulation formation and of their antibiotic resistance levels may improve our understanding of heteroresistance as a cause of clinical treatment failure^15^. Experiments shown in Fig. 4 provide evidence that subpopulation formation under *opvAB* control allows accurate modulation of the number of resistant cells present in a population. In principle, the method should be applicable to any antibiotic resistance gene. Because we were able to specifically vary the frequency of resistant bacteria in the population, this approach provides a proof of concept to study how different frequencies of resistant subpopulations may affect the outcome of antimicrobial treatment *in vivo* (e. g., in a murine model). In theory, mixing constitutively resistant and susceptible strains that are otherwise isogenic would also lead to bacterial cultures with pre-defined amounts of resistant bacteria. However, to reach specific frequencies of resistant bacteria our OpvAB-based approach requires mixing bacteria at frequencies 100-fold lower (e. g., to reach frequencies of 1 × 10^−6^ Km^r^ resistant bacteria, the P_opvAB_::BI-*aac3* strain was mixed at a frequency of 1 × 10^−4^). Thus, an advantage of our *opvAB* switch-based approach is that it can be expected to be less affected by infection bottlenecks that could otherwise eliminate very small subpopulations of bacteria present in the inoculum^21^. For example, one such bottleneck is observed during cecum colonization by *Salmonella* in mice 2–4 days after oral infection, and is dependent on the inflammatory response induced by *S*. *enterica* invading epithelial cells^22,23^.

Additional applications of the *opvAB* switch can be envisaged, including the design of bistable biosensors. For instance, a strain harboring an P_*opvAB*_::*gfp* fusion might be useful to detect bacteriophages in environmental samples using flow cytometry^24,25^, and to identify DNA methylation inhibitors in screens for novel antimicrobial drugs^26,27^. Sensors of this kind can be expected to be selective as growth will occur under specific circumstances only. Furthermore, use of fluorescence to monitor growth of ON cells can be expected to be sensitive and rapid, and constitutive expression may contribute to robustness, avoiding the problem of instability of transcription-based gene circuits^28^. Besides biosensor design, formation of phenotypic subpopulations under epigenetic control might have additional applications in synthetic biology: for instance, division of labour between subpopulations performing distinct segments of a catabolic pathway might optimize biodegradation processes^29^.

## Methods

### Strains and strain construction

Strains of *Salmonella enterica* serovar Typhimurium and *Escherichia coli* used in this study are listed in Table 1. Strain construction by targeted gene disruption was achieved using plasmids pKD3, pKD4 or pKD13 as templates to generate PCR products for homologous recombination^30^. Antibiotic resistance cassettes introduced during strain construction were excised by recombination with plasmid pCP20^30^. Primers used in strain construction are shown in Table 2. For the construction of translational *lac* fusions on the *S*. *enterica* chromosome, FRT sites generated by excision of Km^r^ cassettes were used to integrate plasmid pCE40^31^. For construction of fluorescent fusions, a DNA fragment containing a promoterless green fluorescent protein (*gfp*) gene and a chloramphenicol resistance cassette was PCR-amplified from plasmid pZEP07^32^, and the resulting PCR product was integrated into the chromosome of each strain. For construction of strains that carry antibiotic resistance genes under P_*opvAB*_ control, a counterselectable cassette containing *sacB* and Ap^R^ genes was amplified from strain DA52596 using the oligos opvAB-ampsacB-F and ampsacB-gfp-R. The PCR product was integrated into the chromosome of SV6727 and SV6729 respectively, generating the intermediate strains MN441 and MN442, respectively. Antibiotic resistance genes were introduced into these strains by targeted gene disruption^30^, and transformants in which the ampicillin-*sacB* cassette had been excised were selected on minimal plates containing sucrose.

**Table 1:** Bacterial strains and plasmids.

Strain	Genotype
ATCC 14028^a^	Wild type
MG1655^b^	Wild type
CC118 λ *pir*^b^	*phoA20 thi-1 rspE rpoB argE(Am) recA1 (*lambda *pir*)
S17 λ *pir*^b^	*recA pro hsdR* RP4-2-Tc::Mu-Km::Tn7 (lambda *pir*)
DA52596^a^	*∆(CRISPR1-cas1)::spc ∆(CRISPR2)::amp-sacB / pSim5-tet*
SV6727^a^	*opvAB::gfp*
SV6729^a^	*opvAB* GATC-less::*gfp*
SV8499^a^	*P*_*opvAB*_::*lacZY*
SV9700^a^	*P*_*opvAB*_::*lacZY*::*gfp*
SV9701^a^	P_*opvAB*_ GATC_1,2_::*lacZY*::*gfp*
SV9702^a^	P_*opvAB*_ GATC-less::*lacZY*::*gfp*
SV9703^a^	P_*opvAB*_::BI-*aac6*::*gfp*
SV9705^a^	P_*opvAB*_ GATC-less::BI-*aac6*::*gfp*
SV9706^a^	P_*opvAB*_::BI-*ctxm*::*gfp*
SV9707^a^	P_*opvAB*_ GATC-less::BI-*ctxm::gfp*
DR3^a^	∆*lacZY*
DR22^b^	∆*lacZY* P_*opvAB*_::*lacZY*::*gfp*
DR23^b^	∆*lacZY* P_*opvAB*_ GATC_*1*,*2*_::*lacZY::gfp*
DR24^b^	∆*lacZY* P_*opvAB*_ GATC-less::*lacZY*::*gfp*
MN441^a^	P_*opvAB*_::*bla-sacB*::*gfp*
MN442^a^	P_*opvAB*_ GATC-less::*bla-sacB::gfp*
SV9776^a^	P_*opvAB*_::BI-*aac3*::*gfp*
SV9777^a^	P_*opvAB*_::*gfp*
Plasmid	Description
pIZ2224	pDMS197::P_*opvAB*_ GATC_*1*,*2*_
pIZ2234	pDMS197::P_*opvAB*_ GATC-less

^a^*S. enterica*; ^b^*E*. *coli*.

**Table 2:** Oligonucleotides.

Name	Sequence (5′-3′)	Use
Operonlac-PS1	ATGATAGCGCCCGGAAGAGAGTCAATTCAGGGTGGTGAATGTGTAGGCTGGAGATGCTTC	Amplification of Km^r^ gene from pKD4
Operonlac-PS2	TAGGCCTGATAAGCGCAGCGTATCAGGCAATTTTTATAATCATATGAATAT	
MG1655-opvA	ATGATAGCGCCCGGAAGAGAGTCAATTCAGGGTGGTGAATTCATTTGGTTATAAATAGAG	Amplification of the *opvAB* operon (wild type and variants)
MG1655-opvB	TAGGCCTGATAAGCGCAGCGTATCAGGCAATTTTTATAATGAGTTTATCTCTGCGCAATGT	
pCE40lacY-gfp-5	GCTTTCCCTGCTGCGTCGTCAGGTGAATGAAGTCGCTTAATAAGAAGGAGATATACATATGAG	Amplification of *gfp* and Cm^r^ genes from pZEP07
pCE40-km-3	AAACTGTCTGCTTACATAAACAGTAATACAAGGGGTGTTTTATCACTTATTACAGGCGTA	
opvA-aac6-F	TCTTATGAAGAAATATACGTTCGCTAAGGAGGTTTTCTAATGAGCAACGCAAAAACAAAG	Amplification of the *aac(6*′*)-Ib-cr* gene
aac6-gfp-R	AAAGTTCTTCTCCTTTACTCATATGTATATCTCCTTCTTATTAGGCATGACTGCGTGTTC	
opvA-ctxm-F	TCTTATGAAGAAATATACGTTCGCTAAGGAGGTTTTCTAATGGTTAAAAAATCACTGCG	Amplification of the *bla*_*CTX-M-15*_ gene
ctxm-gfp-R	AAAGTTCTTCTCCTTTACTCATATGTATATCTCCTTCTTATTACAAACCGTCGGTGACGA	
PopvAB-aac3-F	ATCTTATGAAGAAATATACGTTCGCTAAGGAGGTTTTCTAATGCATACGCGGAAGGCAATAAC	Amplification of the *aac3-Ib* gene
GFP-aac3-R	GAAAAGTTCTTCTCCTTTACTCATATGTATATCTCCTTCTTACTAACCGGAAGGCTCGCAAG	
opvAB-ampsacB-F	TCTTATGTGTGGGTTTTATCTTATGAAGAAATATACGTTCGCTAAGGAGGTTTTCTAATGTAGGCTGGAGCTGCTTC	Amplification of the *bla-sacB* cassette
ampsacB-gfp-R	AAAGTTCTTCTCCTTTACTCATATGTATATCTCCTTCTTACATATGAATATCCTCCTTAGTTCC	

Transductional crosses using phage P22 HT 105/1 *int201*^33^ were used for transfer of chromosomal markers between *S*. *enterica* strains^34^. To obtain phage-free isolates, transductants were purified by streaking on green plates^35^. Phage sensitivity was tested by cross-streaking with P22 H5.

Directed construction of point mutations was achieved using the QuikChange^®^ Site-Directed Mutagenesis Kit (Stratagene) using the suicide plasmid pDMS197^36^ and propagated in *E*. *coli* CC118 λ *pir*. Plasmids derived from pMDS197 (pIZ2224 and pIZ2234) were transformed into *E*. *coli* S17-1 λ *pir*. The resulting strains were used as donors in matings with *S*. *enterica* SV9700, selecting tetracycline-resistant transconjugants on minimal plates. One transductant from each mating was propagated as strains SV9701 and SV9702.

### Culture media and growth conditions

Bertani’s lysogeny broth (LB)^37^ was used as standard liquid medium. Solid LB contained agar at 1.5% final concentration. Mueller-Hinton (MH) broth and agar^38^ was used in antibiotic susceptibility tests. Green plates^35^ contained methyl blue (Sigma-Aldrich) instead of aniline blue. The indicador of β-galactosidase activity in plate tests was 5-bromo-4-chloro-3indolyl-β-D-galactopyranoside (X-gal; Sigma-Aldrich, 40 mg/L). No-carbon essential (NCE) medium^39^, supplemented with either glucose (0, 2%) or lactose (0, 2%), was used as minimal medium. When necessary, antibiotics were added to the culture medium at following concentrations: ampicillin (100 mg/L), kanamycin (50 mg/L), chloramphenicol (25 mg/L), and cefotaxamine (40 mg/L).

### Flow cytometry

Bacterial cultures were grown in LB at 37 °C until exponential phase (O.D._600_ 0.3). Cells were then diluted in PBS to a final concentration of approximately 10^7^/ml. Data acquisition and analysis were performed using a Cytomics FC500-MPL cytometer (Beckman Coulter). Data were collected for 100 000 events per sample, and were analyzed with CXP and FlowJo8.7 software. Data are represented by a dot plot (forward scatter [cell size] versus fluorescence intensity.

### Growth curves

Plates were incubated at 37 °C with shaking on an automated microplate reader (Synergy HTX Multi-Mode Reader, Biotek), and the absorbance at 600 nm for each well was measured every 30 min. Each sample was assayed by triplicate. Growth of SV9700, SV9701, SV9703, DR22, DR23 and DR24 strains was monitored in NCE-lactose and NCE-glucose. Growth of SV9704, SV9705, SV9706, SV9707 was monitored in LB broth with and without antibiotics.

### Calculation of phase variation rates

Phase variation rates were estimated as described by Eisenstein^40^. Briefly, a strain harboring a *lacZY* fusion was plated on LB + X-gal and colonies displaying an ON or OFF phenotype after 16 h growth at 37 °C were selected, resuspended in PBS and re-spread on fresh LB + X-gal plates. Phase variation frequencies were calculated using the formula *(M/N)/g* where *M* is the number of cells that underwent phase variation, *N* the total number of cells, and *g* the total number of generations that gave rise to the colony.

### Epsilometer (E) tests of antibiotic resistance

Etest strips were purchased from bioMérieux. Mixtures of overnight cultures of bacteria grown in MH broth were diluted 1:25 in phosphate buffered saline (PBS) to reach cell densities of 0.5 MacFarland or about 1.5 × 10^8^ CFU/mL. Bacteria were plated onto MH agar plates using sterile cotton swabs dipped in the cell suspensions, and a Etest strip was applied on top. Plates were incubated 18 h at 37 °C before reading the results and taking pictures.

### Population analysis profile (PAP) tests

PAP tests were performed on MH agar plates supplemented with increasing amounts of kanamycin (Sigma Aldrich) as described previously^15^. Five µl of overnight cultures in MH broth (containing approx. 3 × 10^9^ cells/ml) and serial dilutions (down to 10^−6^) were spread on MH plates containing no antibiotics (for total CFU determination) or different concentrations of kanamycin. The plates were incubated overnight and the colonies were counted. Colony numbers were plotted in a graph to determine if the PAP fulfilled the criteria for heteroresistance (at least 8-fold difference in antibiotic concentration between the highest non-inhibitory concentration and the highest inhibitory concentration).

To prepare mixtures of resistant and susceptible cells, three isolated colonies of SV9776 (P_*opvAB*_::BI-*aac3*::*gfp*, kanamycin resistant in the ON state) and SV9777 (P_*opvAB*_::*gfp*, always kanamycin susceptible) were grown overnight in 2 mL MH broth at 37 °C under shaking. Pure cultures of each overnight or three independent sets of SV9776:SV9777 mixtures at proportions ranging from 1:10 to 1:10,000 were used for PAP tests.
