# Publisher Correction: A portable epigenetic switch for bistable gene expression in bacteria

**DOI:** 10.1038/s41598-019-54657-2

**Published:** 2019-11-25

**Authors:** David R. Olivenza, Hervé Nicoloff, María Antonia Sánchez-Romero, Ignacio Cota, Dan I. Andersson, Josep Casadesús

**Affiliations:** 1 Departamento de Genética, Facultad de Biología, Universidad de Sevilla, Apartado 1095, 41080 Sevilla, Spain; 2 Department of Medical Biochemistry and Microbiology, Uppsala University, 751 23, Uppsala, Sweden; 3 Present Address: Centre for Research in Agricultural Genomics, CSIC-IRTA-UAB-UB, Campus UAB, Bellaterra, 08193 Barcelona, Spain

Correction to: *Scientific Reports* 10.1038/s41598-019-47650-2, published online 02 August 2019

In the original version of this Article, the author María A. Sánchez-Romero was incorrectly indexed. This error has now been corrected.

